# Unlocking the Complexity of Antibody-Drug Conjugates: A Cutting-Edge LC-HRMS Approach to Refine Drug-to-Antibody Ratio Measurements with Highly Reactive Payloads

**DOI:** 10.3390/ijms26073080

**Published:** 2025-03-27

**Authors:** Andrea Di Ianni, Kyra J. Cowan, Federico Riccardi Sirtori, Luca Barbero

**Affiliations:** 1 Molecular Biotechnology Center, Department of Molecular Biotechnology and Health Sciences, University of Turin, 10126 Turin, Italy; 2 NBE-DMPK Innovative BioAnalytics, Merck RBM S.p.A., an affiliate of Merck KGaA, Darmstadt, Germany, Via Ribes 1, 10010 Colleretto Giacosa (TO), Italy; federico.riccardi-sirtori@merckgroup.com; 3 New Biological Entities, Drug Metabolism and Pharmacokinetics (NBE-DMPK), Research and Development, Merck KGaA, Frankfurterstrasse 250, 64293 Darmstadt, Germany; kyra.cowan@merckgroup.com

**Keywords:** liquid-chromatography mass spectrometry, drug discovery, drug-to-antibody ratio, antibody–drug conjugates

## Abstract

The complexity of therapeutic proteins like antibody–drug conjugates (ADCs) holds a tremendous analytical challenge. Complementary mass spectrometry approaches such as peptide mapping and intact mass analysis are required for the in-depth characterization of these bioconjugates. Cysteine-linked ADCs have shown a unique challenge for characterization, mainly when the conjugation is carried out on interchain cysteines, because their intact analysis requires native mass spectrometry conditions to preserve non-covalent binding between antibody chains. In this work, two different approaches were proposed. Specifically, a full scan data-independent all ion fragmentation (FS-AIF) and a full scan data-dependent targeted MS2 (FS-ddtMS2) were applied to generate complementary datasets for a cysteine-linked ADC characterization with a highly reactive payload. These two methods were applied to in vitro plasma stability and in vivo PK samples to calculate and refine mean drug-to-antibody ratio over time. Using this approach, we successfully characterized an ADC containing a hydrolysis-sensitive payload and refined the “active” drug-to-antibody ratio on in vitro stability and in vivo samples. These two methods allowed the confirmation of the different ADC species and potential metabolites of conjugated payload attached to the antibody backbone in a single analysis without needing a dedicated method for the conjugated payload metabolite identification.

## 1. Introduction

Targeted therapy is a type of cancer treatment that lays the foundation for precision medicine by targeting proteins that control how cancer cells divide, grow, and propagate. To make this feasible, a selective “carrier” must be required to transport the active molecule to the target site only on the tumor cells. Drug development companies have been improving their design of better tumor-targeted therapies using small molecules conjugated to monoclonal antibody carriers [1,2]. The selective delivery of pharmacologically active drugs to target tissues or cells by antibodies (Abs) makes this immuno-conjugate a promising modality for different cancer treatment [3,4,5]. Consequently, the targeted delivery of highly potent cytotoxic drugs in antibody–drug conjugates (ADCs) has improved the selective “druggability” of several tumor antigens, resulting in minimal systemic toxicity and offering patients the benefits of cancer treatment with less severe side effects [6]. Currently, over 100 ADC drugs are in various stages of development worldwide. Among them, 14 have been approved for marketing, a dozen has entered clinical phase III, while the majority are still in clinical phase I and preclinical stages [7]. Drug conjugation can generally be achieved via reactions at lysine ε-amine side chain or cysteine sulfhydryl groups after the reduction in the interchain disulfide bonds [8,9,10]. Recently, ADC bioconjugation has also been achieved through chemoenzymatic-based conjugation (with glycosyltransferase, transglutaminase or transpeptidase [11,12,13]) or site-specific conjugation using non-natural amino acids [14,15,16]. Non-cleavable linkers provide high stability in the bloodstream. They rely on the internalization of the ADC into the target cells, followed by lysosomal delivery and degradation of the ADC complex to release the active drug and kill cancer cells [17]. They may not release drugs into the extracellular space and cannot kill neighboring tumor cells through the bystander effect [18]. Furthermore, optimal linker selection depends on the chosen target antigen. Protease-cleavable linkers are also designed to be stable in plasma, but rapidly release the active drug within lysosomes in cancer cells upon cleavage by lysosomal enzymes [19]. They take advantage of the high levels of protease activity inside lysosomes and include a peptide sequence recognized and cleaved by these proteases. The primary goal of all linkers in ADCs is to ensure the specific release of the active drug within target cells, which is essential for controlling the toxicity of the highly potent drugs used to construct ADCs. However, the balance between efficacy and toxicity changes for the above-mentioned linkers. Moreover, the ideal linker chemistry choice ultimately depends on experimentally determining the optimal combination of the suitable linker, the ideal target antigen, and the desired payload [20,21]. A comprehensive understanding of the structural integrity of the linker, the payload and the conjugation site during biological exposure is crucial throughout the process of novel linker-payload design and optimization of the PK profile [22]. This understanding represents a key aspect in maximizing efficacy while minimizing toxicity in preclinical testing and it is essential for ensuring the successful translation to the clinical setting [23,24].

**Figure 1:**
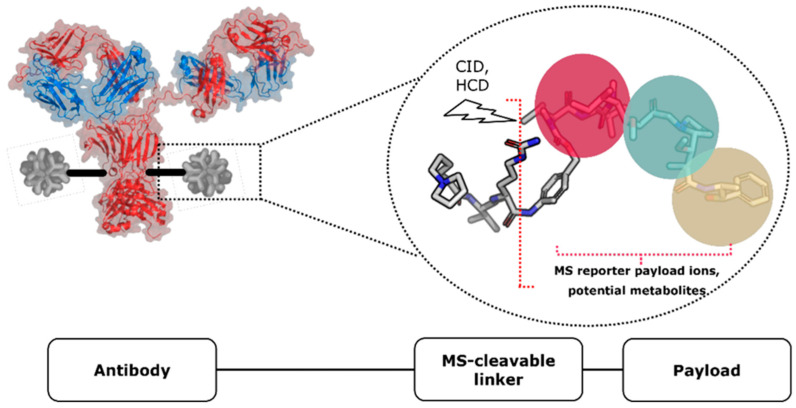
Schematic structure of an antibody–drug conjugate (ADC) and potential strategies for mass spectrometry characterization. The most common strategy is to characterize ADCs via intact protein or bottom-up LC-MS analyses, making it harder to identify antibody-conjugated payload metabolites (MS1-only strategy). An alternative strategy can be applied by taking advantage of the cleavability feature of the linker. This approach promotes the formation of payload fragments in the gas phase during mass spectrometry analysis. By triggering MS2 fragmentation, this method enables the potential identification of small molecule metabolites derived from the ADC payload.

This bioconjugate modality’s complexity is a source of significant challenge for analytical investigation and analysis, especially in vivo [25]. Nowadays, both Enzyme-linked Immunosorbent Assay (ELISA) and Liquid Chromatography coupled to mass spectrometry (LC–MS) have been employed to support ADC discovery and development projects [26,27]. Ligand-binding assays (LBAs) are considered the golden standard to measure antibody concentration (both total and conjugated antibody analytes), whereas LC-MS methods are usually employed to quantify both free and conjugated toxin [28,29]. However, with the advent of hybrid ligand binding LC-MS methods (LB-LC-MS/MS), mass spectrometry-based assays can also be used to assess total and conjugated antibody concentrations [30]. In vivo/vitro intact protein mean drug-to-antibody ratio (DAR) determination is very useful when the toxin is unstable in plasma. Using this method, it is possible to monitor the stability of the linker-payload system without any cleavage or digestion step. In addition, this method can include an anti-human IgG affinity capture. Therefore, it can be adapted to human derived ADCs without any method setup [31]. For this reason, it is possible to apply the affinity capture-MS method (AFC-MS) in the early phases of ADC development, while other assays, such as conjugated payload, are about to be developed in parallel [32]. Therefore, data from the three methods must be combined to infer the overall ADC behavior. For instance, mean DAR variation represents an essential parameter for evaluating ADCs. It reflects the amount of conjugated payload that still has the potential to selectively hit the biological target, and it is therefore responsible for the ADC mechanism of action. The DAR can be indirectly calculated by taking the ratio of two independent measurements: the conjugated payload concentration and the total antibody concentration. However, this evaluation is significantly affected by the error propagation from these two individual measurements. For this reason, it is becoming essential to develop multi-attribute methods (MAM) to fully characterize these bioconjugates while minimizing the number of different assays required from health authorities [33]. This paper proposes a new combinatorial approach that takes advantage of the use of high-resolution mass spectrometry (HRMS), both in targeted data-dependent acquisition (DDA) and data-independent (DIA) full-scan MS2 acquisition strategies (Figure 1).

Using this dual approach, it was not only possible to have an accurate identification of different ADC species in in vitro and in vivo mouse plasma samples, but also to characterize in MS/MS the small molecule payload that is attached to the antibody by exploiting the MS-cleavability bonds of the linker. Eventually, the MS2 strategy can reinforce the identified species proposed by solely looking at intact MS1 level, and it can give more information about the potential metabolic liabilities of the conjugated payload. In fact, modifications of the payload structure could make it inactive or less active from a pharmacodynamic point of view. Therefore, this could be consistent with further characterization of conjugated linker payload via bottom-up LC-HRMS, or more standard antibody-conjugated toxin assays with low-resolution MS analyzers. Finally, this approach can generally be used to refine the in vitro/in vivo mean DAR determination by intact protein LC-MS analysis for ADCs with unstable payloads, further accelerating the development of such compounds to treat human diseases.

## 2. Results and Discussion

### 2.1. Intact Denaturing Non-Covalent ADC Analysis: An ADC Case Study with a Hydrolysis-Sensitive Payload

**Figure 2:**
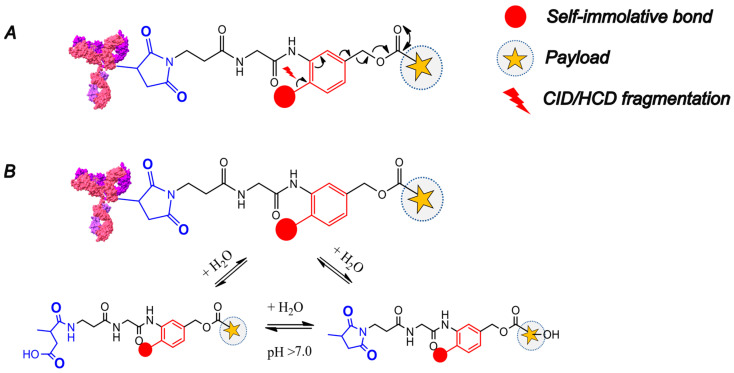
(**A**) HCD/CID-based linker fragmentation pattern in mass spectrometer Ion Routing Multiple for ADC linker-payload system. (**B**) Potential spots on linker-payload that are sensitive to hydrolysis in plasma. The succinimide ring is highlighted in blue. Linker payload is conjugated on interchain cysteines (one linker payload is shown for simplification).

ADC-A is a DAR 4 non-covalent ADC conjugated on interchain cysteines with a payload potentially sensitive to hydrolysis, which could make it inactive. The conjugation is carried out via reduction in interchain disulfide bonds and maleimide-based linker payload chemistry. In principle, the light chain of the ADC could have up to max 1 linker-payload. Instead, the heavy chain could be conjugated up to three linker payloads. The hydrolysis modification on the linker payload system can occur on the succinimide ring or on the payload that might hydrolyze in plasma. This results in the same monoisotopic mass modification on the ADC intact mass of +18.0106 Da (Figure 2).

**Figure 3:**
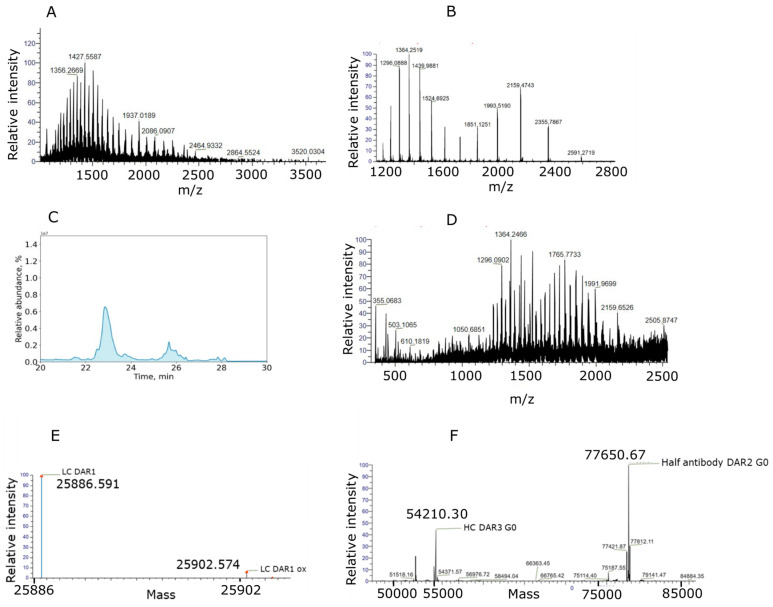
Total Ion Chromatogram for conjugated LC and HC. (**A**) ADC-A glycosylated Heavy chain MS spectrum at 0 h in mouse plasma. (**B**) ADC-A light chain MS spectrum at 0 h in mouse plasma. (**C**) Total Ion chromatogram of separated conjugated HC (R_T_ = 21.8 min), LC (R_T_ = 23.40 min) and half-antibody (R_T_ = 25.84 min) of ADC-A at 0 h in mouse plasma in vitro stability. (**D**) ADC-A glycosylated half-antibody MS spectrum at 0 h in mouse plasma. (**E**) Deconvoluted spectrum of ADC-A light chain MS spectrum at 0 h in mouse plasma. (**F**) Deconvoluted spectrum of ADC-A heavy chain and half-antibody MS spectrum at 0 h in mouse plasma.

The observed water addition must be correctly allocated to either the succinimide ring of the linker (and/or on the antibody backbone) or potentially on the payload itself. Incorrectly assigning the site of hydrolysis can completely change the “real” active DAR evaluation, potentially leading to a misinterpretation of the ADC’s behavior. In addition, potential modifications on the protein backbone (e.g., oxidation, deamidation and retro-Micheal adducts) must be considered. Leveraging the cleavable bond feature of the linker, which is crucial for the ADC’s mechanism of action, we used a collisional-induced dissociation/Higher-energy collisional induced dissociation (CID/HCD)-based fragmentation strategy to induce the formation of the payload ion fragments from the antibody-linker precursor ions, thus identifying potentially hydrolyzed antibody-conjugated payload species. In Reversed Phase Liquid Chromatography (RPLC), the non-covalent interactions are no longer conserved and then the ADC breaks down into its single chains, the conjugated light chain (LC) and heavy chain (HC) as depicted in Figure 3. For this ADC, also half-antibody species were identified.

### 2.2. MS2-Based Identification Strategy Enables Better Understanding of ADC Conjugated with Highly Reactive Payloads

**Figure 4:**
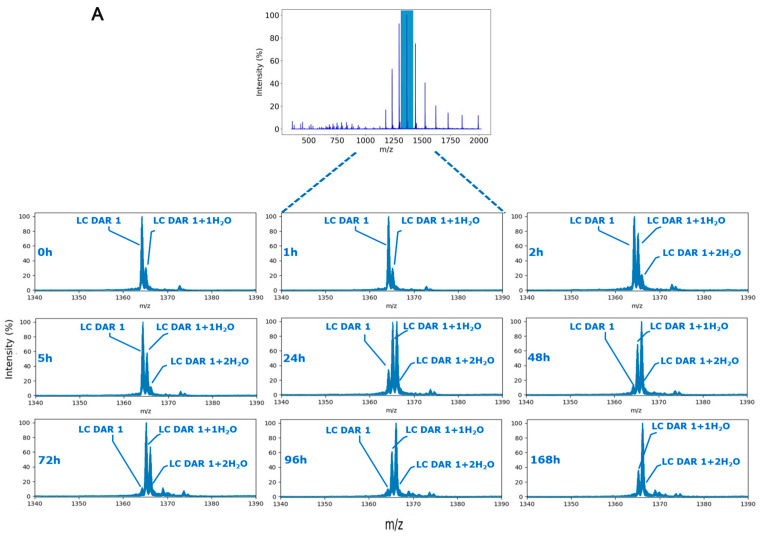

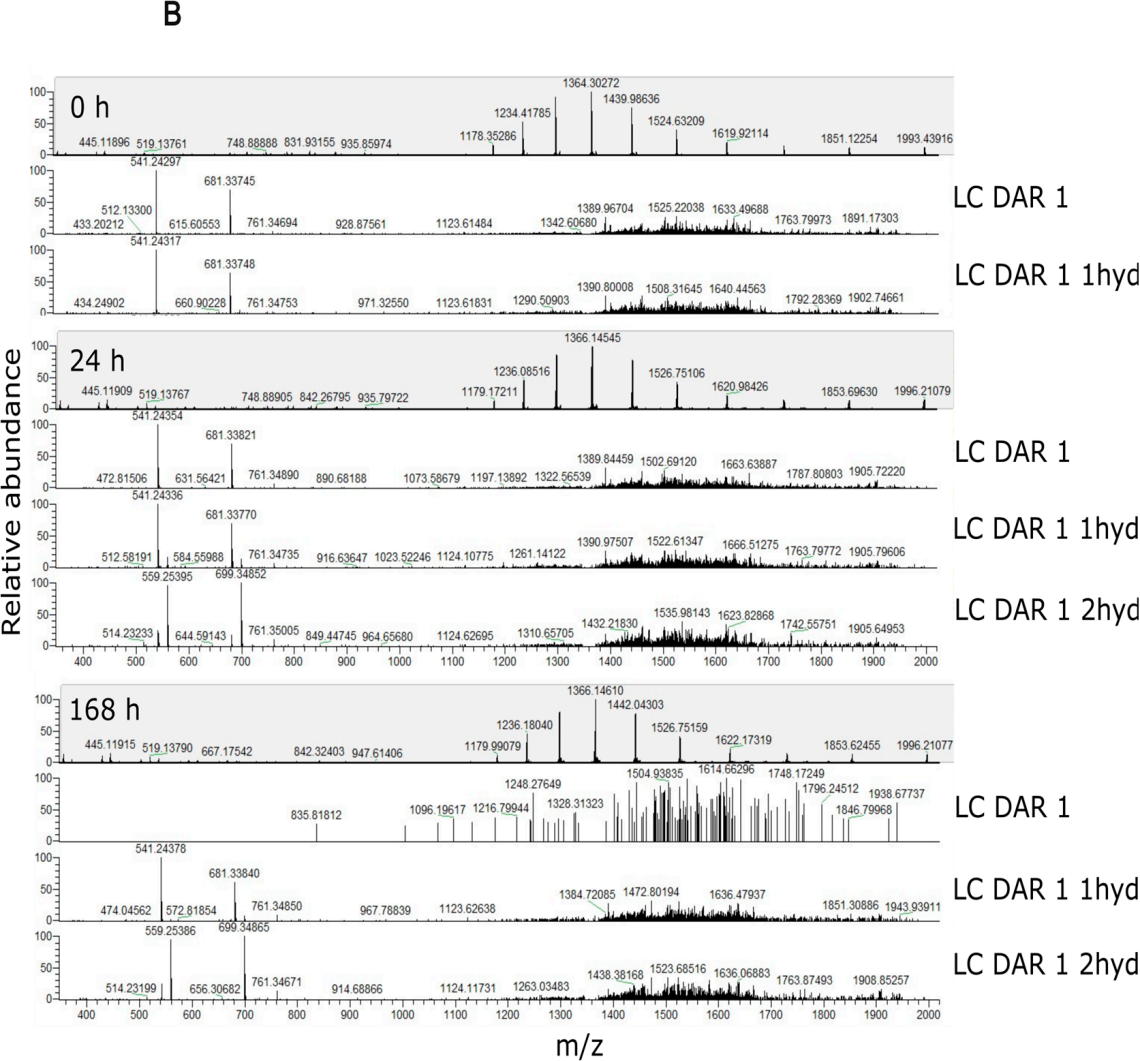
Hydrolysis rate evaluation for ADC-A LC-conjugated chain. (**A**) MS1 spectra of different in vitro mouse plasma stability samples, zooming around *m/z* 1364.25, corresponding to the unmodified LC DAR 1 ADC species. (**B**) Targeted-MS2 fragmentation ion mass spectra for the three identified LC-conjugated payload species in the survey MS1 scan used to calculate the payload hydrolysis rate. From 24 h on, the hydrolyzed antibody-conjugated payload species started to appear (mainly in bi-hydrolyzed species).

A first survey full-scan MS acquisition was used to analyze in vitro mouse plasma stability samples of ADC-A after separating its chains in RPLC. MS1-only identification strategy is then normally applied to calculate ADC mean DAR over time. This could lead to potential misinterpretation of the ADC behavior, especially when small modifications (e.g., hydrolysis) occur on the conjugated payload, according to the resolution of the MS method. In this case, when a single hydrolysis occurs (corresponding to a mass modification of +18.0106 Da), the assumption of having it on the succinimide ring of the linker was supposed. On the other hand, a double hydrolyzed species (mass modification of +36.0212 Da) was assigned on succinimide and payload, respectively (the protein backbone was not considered because hydrolysis is not reported in ADC literature to impact the amino acid protein backbone). After tentatively assigning the different species by MS1-only acquisition, a second run of targeted MS2 was acquired on the same in vitro stability samples to selectively isolate and confirm previously identified hydrolyzed species by MS1-only analysis. Basically, a mass-targeted inclusion list of MS1-identified species was included to trigger the MS2 experiments specifically (see Figure 4A, Table S1). In this way, the cleavability feature of the linker was used to promote the formation of the payload ions in gas-phase to identify the hydrolysis site. Two main fragments from the payload were present in MS2 spectra (respectively, *m/z* 541.24 and 681.34 and their respective hydrolyzed species, Figure 4B). From the total extracted ion chromatograms (XICs) of the payload fragments, the mean percentage of hydrolyzed payload was calculated in respect to the total amount of detected payload related ions (neat+ hydrolyzed payload, Equation (1) and Figure 4A,B).

(1) $$mean\;\%\;of\;hydrolyzed\;payload=\sum_{n}^{i}\frac{Total\;chain\;hydrolyzed\;payload\;XIC}{Total\;chain\;hydrolyzed+neat\;payload\;XIC}\times 100$$

Equation (1): equation for calculating the percentage of total hydrolyzed payload on light/heavy chain.

**Figure 5:**
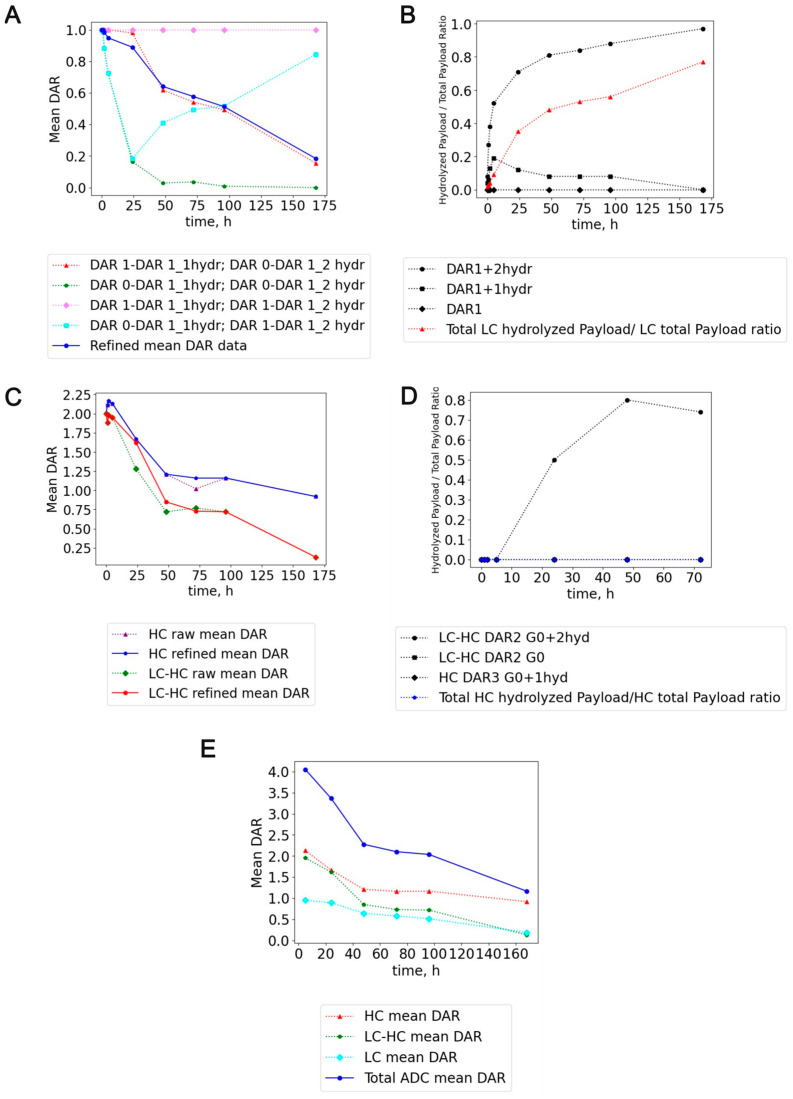
Different DAR calculation hypotheses impact and comparison to actual MS2-refined DAR mean value for ADC-A in vitro mouse plasma stability samples. (**A**) ADC-A MS2-refined LC DAR analysis. LC can be conjugated up to 1 linker payload (LC DAR 1). LC DAR 0 is the unmodified light chain or LC with inactive hydrolyzed payload. Different hypotheses for DAR calculation can be made in advance. Red trace considered mono-hydrolyzed LC species as fully “active” species (e.g., DAR1 + 1 hydrolysis, namely DAR1_1hydr) and bi-hydrolyzed species as fully “inactive” species (e.g., DAR1 + 2 hydrolysis, DAR1_2hydr, assigned as DAR0). Green trace considered mono and bi-hydrolyzed species as inactive species (e.g., both DAR1_1hydr and DAR1_2hydr species as DAR0, then considering the payload more hydrolysis-sensitive than succinimide ring linker). Purple trace considered mono and bi-hydrolyzed species as active species (e.g., both DAR1_1hydr and DAR1_2hydr species as DAR1). Cyan trace is an unrealistic scenario of having mono-hydrolyzed species as fully inactive (e.g., DAR0) and bi-hydrolyzed species as fully active (e.g., DAR1). The blue trace is the experimentally MS2-refined DAR value for ADC-A conjugated LC using targeted DDA MS/MS experiments. (**B**) Hydrolyzed over total payload ratio for the mono and bi-hydrolyzed LC DAR1 species. The red line represents the average total hydrolyzed payload (mono + bi-hydrolyzed LC DAR1 species) over the total payload ratio for ADC-A LC. (**C**) ADC-A MS2-refined HC and half-antibody DAR analysis. (**D**) Hydrolyzed over total payload ratio for the identified mono-hydrolyzed HC DAR3 G0 and bi-hydrolyzed glycosylated half-antibody (LC-HC DAR2 G0 2hyd). Black and blue lines represent the total hydrolyzed payload over the total payload ratio for ADC-A half-antibody and HC, respectively. (**E**) Total ADC DAR and refined mean DAR of individual ADC chains.

With this targeted MS2 strategy, water addition on the payload attached to the antibody was readily detected (Figure 4B) and DAR value was refined (Figure 5A,C,E). Depending on the initial hypothesis, MS1-only strategy can lead to different ADC DAR values, depending on where the hydrolysis is assigned (Figure 5A). Interestingly, the hydrolysis started to be consistent from 24 h on the light chain (35% hydrolysis rate from mono and bi-hydrolyzed LC DAR1 species) and reached the maximum up to 168 h (77% hydrolysis rate, Figure 5B). Conversely, the heavy chain was utterly protected from payload hydrolysis. Moreover, half-antibody ADC species were also identified, having a discrete hydrolysis rate (more than 70% of hydrolysis rate at 48 h for the glycosylated bi-hydrolyzed half-antibody species, Figure 5D).

**Figure 6:**
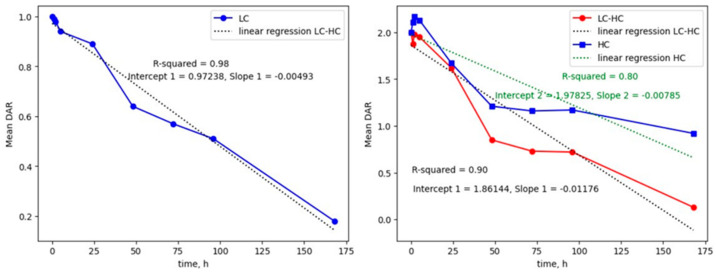
DAR decay regression analysis of ADC-A in in vitro mouse plasma stability. DAR loss over time regression analysis for LC (**left**) and HC and half-antibody (**right**) ADC species, slope represents the DAR loss unit/h in the time frame of in vitro mouse plasma stability at 37 °C and 5% CO_2_.

Based on our findings, DAR loss from light chain can be primarily imputed to the hydrolysis of conjugated payload on the ADC, thus making it inactive (namely a DAR-impacting modification, Figures S1–S3 and S6, and Table S2). On the other hand, heavy chain was more shielded from hydrolysis, but it showed a high sensitivity relating to retro-Micheal reaction in mouse plasma (Figures S4, S5, S7 and Table S3). As it can be seen from Figure 5A, depending on the initial hypothesis, different DAR values can be obtained using an MS1-only intact ADC LC-HRMS approach. Our supposed hypothesis was to assign a “full” DAR value for mono-hydrolyzed species and a “DARn-1” value for bi-hydrolyzed ADC LC species. Then, we considered the antibody-conjugated payload less sensitive to hydrolysis in plasma than the succinimide ring linker (whose hydrolysis can even stabilize ADC to retro-Micheal reaction). This hypothesis brought to DAR estimation was the closest to the MS2-refined DAR values at all different in vitro mouse plasma stability timepoints. Other optimistic hypotheses (mono and bi-hydrolyzed species assigned as an entirely active DAR species) or more cynical views (both mono and bi-hydrolyzed species assigned as DARn-1 species) were far from the MS2-refined DAR values. Moreover, the analysis of the DAR profile over time showed that both chains followed a pseudo-linear decay. From the regression line, LC showed a DAR decay of 0.005 units/h. HC, and the half-antibody showed a decay of 0.008 and 0.011 DAR units/h (Figure 6).

This suggests that the retro-Micheal reaction has impacted more on DAR loss than the antibody-conjugated payload hydrolysis. These data highlighted the capability of this approach to locate the hydrolysis and refine the “actual” DAR from the MS1-only hypothesis strategy. Moreover, it seemed the ADC is more protected on the heavy chain from hydrolysis, suggesting that conjugation on heavy chain interchain cysteines may be a good choice for unstable payloads in mouse plasma. Conversely, a trade-off was observed between the number of conjugated payloads on the antibody chain and the occurrence of retro-Micheal reaction.

### 2.3. Method Application on In Vivo PK Mouse Plasma

**Figure 7:**
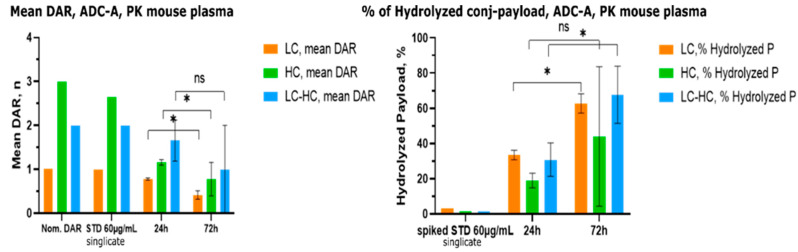
In vivo PK MS2-refined DAR analysis of ADC-A from FS-AIF experiments. (Left) Mean DAR value for the three main ADC conjugated species, LC, HC and half-antibody (*n* = 3) from three C57BL/6N treated mice. Nominal DAR of the chains for this ADC is shown and compared to the ADC standard in buffer (STD in the plot). Bars show 95% confidence interval (2 standard deviations). The asterisk indicates the statistical significance of *p*-value (* corresponds to 95% significance, α = 0.05). (Right) Percentage of hydrolyzed conjugated payload for the three main ADC conjugated species, LC, HC and half-antibody (*n* = 3) from three C57BL/6N treated mice. Bars show 95% confidence interval (2 standard deviations).

To assess the feasibility of this MS2-refined DAR approach, we carried out a single data-independent all-ion fragmentation MS analysis (FS-AIF) analysis on samples collected from a PK study (C57BL/6N mice strain, *n* = 3) performed on ADC-A. Three different ADC conjugated species (LC, HC and half-antibody) were monitored to confirm and correlate our in vitro stability hydrolysis rate data with in vivo findings (Figure 7).

In this case, one-shot FS-AIF analysis was performed due to micro-sampling sample collection which limited the sample volume at disposition for the study. Then, in the MS2 spectrum, the total amount (%) of hydrolyzed payload coming from all different hydrolyzed species extracted ion chromatograms (XICs) in the correspondence to the chromatographic retention time (R_T_) of LC, HC and half-antibody was retrieved from the MS1 spectra survey scan. As can be seen from Figure 7, there is a good agreement between in vitro/in vivo hydrolysis rate for LC and half-antibody conjugated species. In fact, at 24 h ~35% of payload hydrolysis for LC and ~50% of payload hydrolysis for half-antibody in mouse plasma were reported both in vitro and in vivo. For the 72 h timepoint, a good agreement between in vitro stability and in vivo PK mouse plasma samples was also observed (~60% of payload hydrolysis rate for LC and ~70% of payload hydrolysis for half-antibody). Then, similar hydrolysis rates were found between the LC and half-antibody chains at the investigated PK timepoints. Therefore, the hydrolysis of the payload is mainly driven by pH and solvent accessibility, and there are no other in vivo-associated factors. Finally, no other modifications of the antibody-conjugated payload or covalent adducts with plasma proteins were observed.

## 3. Materials and Methods

### 3.1. Chemicals and Reagents

The ADC-A is a DAR 4 ADC and was synthesized at Merck KGaA (Darmstadt, Germany). The Biotin SP-conjugated AffiniPure Goat Anti-human IgG Fc fragment specific capture antibody was purchased at Jackson ImmunoResearch (Product code number:109-065-098, Cambridge House, United Kingdom). PBS tablets were obtained from Life Technologies (18912014, Thermo Scientific, CA, USA). MS grade acetonitrile (ACN), trifluoroacetic acid (TFA), and MS grade water were delivered from Sigma-Aldrich (St. Louis, MI, USA). Streptavidin sepharose magnetic beads were purchased from Cytiva (28985738, GE Healthcare, Marlborough, MA) and Smart Block™ was purchased from Candor Biosciences, (113125, Wangen, Germany). Blank mouse plasma used for the preparation of stability samples was obtained from BioIVT (Westbury, New York, NY, USA).

### 3.2. Preparation of In Vitro Plasma Stability Samples

Stock solution of ADC was spiked in mouse plasma at a final concentration of 140 μg/mL. The resultant plasma samples were incubated in an incubator at 37 °C and 5% CO_2_ for different timepoints (0 h, 1 h, 2 h, 5 h, 24 h, 48 h, 72 h, 96 h, and 168 h). At every timepoint, 50 μL of ADC plasma sample was taken and frozen at −80 °C.

### 3.3. In Vivo PK Study Design

PK study was authorized by Italian Minister of Health. Three C57BL/6N mice were dosed with 23 mg/kg of ADC-A. Micro sampling was used to collect plasma samples (~6 µL of samples for each time PK timepoint, 24 h and 72 h were used for the investigation).

### 3.4. Immuno-Enrichment from Mouse Plasma of Intact ADC-A

120 µL of streptavidin-coated magnetic beads (20.0 mg/mL) were washed three times in PBS and reconstituted in 0.5 mL of assay buffer. Then, 70 µL of Biotin S-SP-conjugated AffiniPure Goat Anti-human IgG Fc fragment specific capture antibody (1.3 mg/mL) were coated on the achieved streptavidin magnetic beads suspension for 2 h on a digital test-tube rotator (Labinco, Breda, Noord-Brabant). Afterwards, the coated beads were washed three other times with PBS and re-suspended in a final volume of 1500 μL. 10 μL of mouse plasma-spiked sample were loaded into a 700 μL Protein LoBind 96-well plate from Waters (Milford, MA, USA). The ADC molecules were enriched by pipetting 150 µL of beads suspension per well in a final volume of 500 µL of PBS and incubated on a Thermomixer at room temperature at 1000 rpm. After the enrichment, samples were rinsed twice with PBS and two times with MS-grade water. Deglycosylation step (overnight, pH 7.4, 37 °C) was skipped to not induce any potential hydrolysis of the payload during sample preparation. Immuno-enriched samples were eluted into clean MaxPeak HPS vials (Waters, MA, USA) using 30 μL of 1% FA in water, Smart Block™ 1:50.

### 3.5. LC-HRMS Method for Non-Covalent Intact ADC Analysis in In Vitro Mouse Plasma Stability Samples

A 5 μL sample of ADC-enriched samples was injected in a full loop configuration into a Dionex UltiMate 3000 LC system coupled to an Orbitrap Eclipse mass spectrometer (Thermo Scientific, Bremen, Germany) on a 5 μL sample loop. The sample was loaded at 5% B onto a MabPac^®^ RP 4 µm, 1500 Å pore size, 0.15 × 150 mm column (Thermo Scientific, PN ES907), that was maintained at 60 °C for the analytical separation. The mobile phases consisted of 0.1% *v/v* HCOOH in water (Phase A) and 0.1% *v/v* HCOOH in ACN (Phase B). The non-covalent ADC-enriched plasma samples were eluted at a flow rate of 1 µL/min, and the following gradient was used: 0.0–4.0 min, 5% B; 4.0–6.0 min, 5–25% B; 6.0–18.0 min, 25–32% B; 18.0–20.0 min, 32–80% B; 20.0–28.0 min, 80% B; 28.0–28.1 min, 80–95% B; 28.1–39.0 min, 95% B; 39.1–50.0 min, 5% B. For MS detection in nanoEasy configuration, a two-segments Full Scan (FS) MS experiments (*m/z* 266.7–4000) was acquired in positive ion mode, without the lock mass option enabled. Specifically, the first Full Scan segment (0–19 min) for the light chain (LC) MS analysis was set to a resolution of 120,000 at full width at half-maximum (FWHM) at *m/z* 200, without any in-source fragmentation, for 5 microscans in Profile mode. A target value of 3.00 × 10^6^ ions was selected for the automatic gain control (AGC) setting with a maximum injection time of 200 ms. The spray voltage was set at 2.0 kV, capillary temperature 275 °C, and S-lens RF level at 60 V. Concerning the second FS segment (19–50 min) for heavy chain (HC) MS analysis, the mass spectrometer resolution was set to 30,000 FWHM at *m/z* 200 for 10 microscans in Profile mode. A target value of 3.00 × 10^6^ ions was selected for the AGC setting with a maximum injection time of 54 ms. The other MS parameters were spray voltage at 2.0 kV, capillary temperature at 275 °C, and S-lens RF level at 60 V.

### 3.6. Targeted Data-Dependent MS/MS Method (tddMS2)

After having assigned all the different ADC species IDs from high-resolution FS MS1 analysis, a second FS targeted data-dependent MS2 (FS-tddMS2) experiment was then applied on in vitro stability samples. Briefly, the most intense ADC species and specific charge states coming from the different identified species (containing unmodified or potentially modified conjugated payload) were selected and a target mass list node was introduced for the MS2 acquisition, by using the Orbitrap quadrupole for different *m/z* isolation (*m/z* 1 isolation window). A CID-based fragmentation strategy with a normalized collisional energy (NCE) of 35 V was applied to enable the fragmentation of MS-cleavable linker to induce the payload ion formation from the ADC in the gas-phase. The list of different m/z targets and proposed ADC species IDs are listed in Supplemental Table S1.

### 3.7. All-Ion Fragmentation MS Analysis (FS-AIF) for In Vivo PK Samples

A single FS-AIF experiment was performed on in vivo PK plasma samples due to limited sample volume. This method has the same survey scan for the intact protein detection, followed by a data-independent all ion fragmentation (AIF) scan, with an HCD fragmentation energy applied (for LC and HC fragmentation, Normalized Collision Energy was set to 30 V), at a resolution of 30,000 FWHM at *m/z* 200 for both chains, maximum injection time of 54 ms, target AGC value of 3.00 × 10^6^ in Centroid mode, with an isolation window of *m/z* 2000 in order to have a single DIA window comprising *m/z* 1000–3000 scan range (in order to let the fragmentation of all different charge states of both antibody chains). In the FS-AIF mode, the instrument continuously acquires two different MS scans. The ions in the first full MS scan are directly injected from the C-Trap into the Orbitrap mass analyzer. Afterwards, the ions of all different charge states of protein envelope in the second DIA scan are sent as a whole into the Ion Routing Multiple (IRM) cell, where they are fragmented. As a result, all product ions and remaining precursor ions are returned to the C-Trap, and then injected into the Orbitrap mass analyzer central electrode.

### 3.8. LC-HRMS Analysis

In vitro stability samples were diluted 1:2 in mouse plasma before immunoaffinity purification with streptavidin coated beads and 10 µL of sample were used. A 5 µL sample of non-covalent cysteine-linked ADC-enriched samples were injected into the LC-MS system. For in vivo PK samples, 24 h and 72 h samples were diluted 1:5 in mouse plasma and 5 µL of the final samples were spiked in 96-well plate for the immunoaffinity step.

### 3.9. Data Analysis

MS spectra deconvolution was performed with the BioPharmaFinder™ software 4.0 (Thermo Scientific) using the Re-Spect™ algorithm for isotopically unresolved spectra with sliding windows for heavy chain analysis. Light chain analysis was carried out using Xtract™ algorithm for isotopically resolved spectra. The DAR species were searched using the linker payload monoisotopic and average masses as variable modifications in the modification editor window of the protein sequence manager as published recently [31]. The input mass range was set from *m/z* 1400 to 5000. The output mass range was restricted to 100–160 kDa, with minimum required adjacent charge states set to 6, whereas the retention time (R_T_) range was restricted to the R_T_ windows of single chains, with an R_T_ tolerance of 1 min from the expected average R_T_. Mean DAR value was calculated using the equation from our previous published work [31]. From the total extracted ion chromatograms (XICs) of the payload fragments, the mean percentage of hydrolyzed payload was calculated (according to Equation (1)) and used to refine the mean DAR over time.

## 4. Conclusions

With the increasing development of ADCs and complex protein constructs—such as bispecific and trispecific antibodies or antibody-VHH constructs—new analytical approaches are needed to address their growing molecular complexity. ADC bioanalysis typically requires the measurement of multiple analytes, including free payload, conjugated payload, and both total and conjugated antibody. As the ADC market rapidly expands, researchers are exploring novel payload chemistries and conjugation strategies to develop ADCs with innovative mechanisms of action, such as targeted-protein degraders, small molecule agonists, or covalent binders. However, for payloads highly reactive with nucleophiles (e.g., protein cysteines or even water), developing conjugated payload quantification methods can be challenging, often requiring derivatization before LC-MS/MS analysis. Furthermore, when using Multiple Reaction Monitoring (MRM) mode on triple-quadrupole mass spectrometers, certain payload adducts or modifications in plasma may be overlooked due to the high specificity of the method. Intact ADC analysis using high-resolution mass spectrometers can help identify intact ADC species (Figures S6 and S7 and Tables S2 and S3). However, when dealing with covalently cysteine-linked ADCs, even high-resolution analyzers like Orbitrap cannot achieve isotopic resolution (especially for antibody heavy chain), and similar small mass modifications on antibody-conjugated payload could be missed or biasedly identified (e.g., antibody-conjugated payload oxidation, hydroxylation, or hydrolysis). To overcome this issue, we developed two approaches (FS-tddMS2 and FS-AIF) that leverage the cleavable nature of the linker-payload system to release the payload attached to the antibody in the gas-phase (simulating what should occur in vivo in lysosomes). By employing these approaches, it becomes possible to achieve monoisotopic resolution on conjugated payload metabolites MS peaks. With these two methods, complementary results can be achieved and can be fine-tuned according to project needs (preferring single FS-AIF analysis over multiple FS-tddMS2 runs when dealing with limited sample volumes from micro-sampling PK studies). In principle, with this MS2-based strategy, every ADC containing unstable payload conjugated with cleavable linkers could be deeply characterized. In addition, the FS-AIF method, which uses a data-independent approach, can achieve better reproducibility and sensitivity compared to data-dependent approaches. Therefore, they should be preferred in PK studies, where total antibody concentrations can be lower compared to in vitro plasma/serum stability studies.

## Data Availability

Data is contained within the article and supplementary material.

## Supplementary Materials

The following supporting information can be downloaded at: https://www.mdpi.com/article/10.3390/ijms26073080/s1.
