# Supplementary material for: Unlocking the Complexity of Antibody-Drug Conjugates: A Cutting-Edge LC-HRMS Approach to Refine Drug-to-Antibody Ratio Measurements with Highly Reactive Payloads
Source: Int J Mol Sci. 2025 Mar 27;26(7):3080. doi: 10.3390/ijms26073080 (PMC11988793; doi:10.3390/ijms26073080)
Supplement: Supplementary file 1 [file ijms-26-03080-s001.zip › ijms-3477787-supplementary.pdf]

Unlocking the Complexity of Antibody-Drug Conjugates: A Cutting-Edge LC-HRMS Approach to Refine Drug-to-Antibody Ratio Measurements with Highly Reactive Payloads

Andrea Di Ianni [1,2,*], Kyra J. Cowan [3], Federico Riccardi Sirtori [2] and Luca Barbero [2,*]

[1] Molecular Biotechnology Center, Department of Molecular Biotechnology and Health Sciences, University of Turin, 10126 Turin, Italy
[2] NBE-DMPK Innovative BioAnalytics, Merck RBM S.p.A., an affiliate of Merck KGaA, Darmstadt, Germany, Via Ribes 1, 10010 Colleretto Giacosa (TO), Italy; federico.riccardi-sirtori@merckgroup.com
[3] New Biological Entities, Drug Metabolism and Pharmacokinetics (NBE-DMPK), Research and Development, Merck KGaA, Frankfurterstrasse 250, 64293 Darmstadt, Germany; kyra.cowan@merckgroup.com
* Correspondence: andrea.diianni@unito.it (A.D.I.); luca.barbero@merckgroup.com (L.B.)

Abbreviations and nomenclature

LC DAR 1 corresponds to conjugated Light chain with 1 payload

LC HC DAR2 G0 corresponds to conjugated half-antibody with 2 payloads, with A2G0 sugar

HC DAR3 G0 corresponds to conjugated heavy chain with 3 payloads, with A2G0 sugar

CID: Collisional-induced dissociation

A2G0: sugar (no fucose, 4 n-acetyl glucosamines, three mannose residues)

A2G1: sugar (no fucose, 4 n-acetyl glucosamines, three mannose residues, 1 galactose residue)

nHyd: number of hydrolysis

1GK loss: Glycine and Lysine C-clipping on Heavy chain C-terminus.

Table S1. Targeted data-dependent CID MS2 inclusion list for ADC Light Chain and Heavy Chain from different ADC species identified from full scan MS1 signals mass spectrometry deconvolution.

Compound	m/z	Time mode	Mass tolerance, ppm
LC DAR 1	1364.17	Unscheduled	25
LC DAR1 hyd	1365.19	Unscheduled	25
LC DAR1 2hyd	1366.2	Unscheduled	25
LC DAR1 2hyd ox	1366.98	Unscheduled	25
LC HC DAR2 G0	1726.56	Unscheduled	25
LC HC DAR2 G0 2hyd	1727.48	Unscheduled	25
HC DAR3 G0	1595.47	Unscheduled	25
HC DAR3 G0 3hyd	1466.62	Unscheduled	25

Table S2. ADC-A LC species identified in *in vitro* stability mouse plasma samples. Hydrolyzed species (from 4’ to 9’) are highlighted in bold.

Species	Species ID
[1]	ADC-A_LC / 1xPayload
[2]	ADC-A_LC / 1xPayload,1xOxidation (MW)
[3]	ADC-A_LC / 1xPayload,1xDeamidation (N),1xOxidation (MW)
[4]	ADC-A_LC / 1xOxidation (MW),1xPayload_1hyd_DAR1
[5]	ADC-A_LC / 1xPayload_1hyd_DAR1
[6]	ADC-A_LC / 1xDeamidation (N),1xOxidation (MW),1xPayload_1hyd_DAR1
[7]	ADC-A_LC / 1xPayload_2hyd_DAR1
[8]	ADC-A_LC / 1xOxidation (MW),1xPayload_2hyd_DAR1
[9]	ADC-A_LC / 1xDeamidation (N),1xOxidation (MW),1xPayload_2hyd_DAR1
**[4’]**	**ADC-A_LC / 1xOxidation (MW),1xPayload_1hyd_DAR0**
**[5’]**	**ADC-A_LC / 1xPayload_1hyd_DAR0**
**[6’]**	**ADC-A_LC / 1xDeamidation (N),1xOxidation (MW),1xPayload_1hyd_DAR0**
**[7’]**	**ADC-A_LC / 1xPayload_2hyd_DAR0**
**[8’]**	**ADC-A_LC / 1xOxidation (MW),1xPayload_2hyd_DAR0**
**[9’]**	**ADC-A_LC / 1xDeamidation (N),1xOxidation (MW),1xPayload_2hyd_DAR0**

Table S3. ADC-A HC and half-antibody species identified in *in vitro* stability mouse plasma samples. *Retro-Micheal species are highlighted in green.*

[1]	ADC-A_HC_G0 / 3xPayload
[2]	ADC-A_HC_G0 / 2xPayload,1xPayload_1hyd
[3]	ADC-A_HC_1GK_loss_G0 / 1xDeamidation (N),3xPayload,1xDecarboxylation
[4]	ADC-A_HC_G0 / 1xOxidation (MW),3xPayload
[5]	ADC-A_HC_1GK_loss_G0 / 2xPayload,1xPayload_1hyd,1xDecarboxylation
[6]	ADC-A_HC_G0 / 1xOxidation (MW),2xPayload,1xPayload_1hyd
[7]	ADC-A_HC_G0 / 1xPayload,2xPayload_1hyd
[8]	ADC-A_LC_HC_G0 / 2xPayload
[9]	ADC-A_LC_HC_G1 / 2xPayload
[10]	ADC-A_LC_HC_G0_1GK_loss / 1xDeamidation (N),1xDecarboxylation,2xPayload
[11]	ADC-A_LC_HC_A1G0 / 2xPayload
[12]	ADC-A_LC_HC_G0 / 1xDeamidation (N),1xPayload,1xPayload_1hyd
[13]	ADC-A_LC_HC_G0 / 1xPayload,1xPayload_1hyd
[14]	ADC-A_LC_HC_1GK_loss_G0 / 1xDeamidation (N),2xPayload
[15]	ADC-A_HC_G0 / 1xDeamidation (N),2xPayload,1xPayload_2hyd
[16]	ADC-A_LC_HC_G0 / 1xPayload,1xPayload_2hyd
[17]	ADC-A_LC_HC_G0 / 1xDeamidation (N),1xPayload_1hyd,1xPayload_2hyd
[18]	ADC-ALC_HC_1GK_loss_G0 / 1xOxidation (MW),2xPayload
[19]	ADC-A_LC_HC_1GK_loss / 2xPayload_1hyd
[20]	ADC-A_HC_G0 / 1xDeamidation (N),1xPayload,1xPayload_1hyd,1xPayload_2hyd
[21]	ADC-A_HC_G0 / 1xOxidation (MW),1xPayload,1xPayload_1hyd,1xPayload_2hyd
[22]	ADC-A_LC_HC_1GK_loss / 2xPayload_2hyd
[23]	ADC-A_HC_G0 / 2xPayload_1hyd,1xPayload_2hyd

[24]	ADC-A_LC_HC_1K_G0 / 1xDeamidation (N),2xPayload
[25]	ADC-A_HC_G0 / 1xDeamidation (N),1xOxidation (MW),1xPayload
[26]	ADC-A_HC_1GK_loss / 1xDeamidation (N),1xOxidation (MW),1xPayload_1hyd
[27]	ADC-A_LC_HC_1GK_loss / 1xDeamidation (N),1xPayload_1hyd,1xPayload_2hyd
[28]	ADC-A_HC_G0 / 1xDeamidation (N),2xOxidation (MW),1xPayload_1hyd
[17']	ADC-A_LC_HC_G0 / 1xDeamidation (N),1xPayload_1hyd,1xPayload_2hyd
[16']	ADC-A_LC_HC_G0 / 1xPayload,1xPayload_2hyd
[29]	ADC-A_HC_G0 / 1xDeamidation (N),1xPayload,2xPayload_2hyd
[30]	ADC-A_LC_HC_G0_1GK_loss / 1xDeamidation (N),1xDecarboxylation,1xPayload,1xPayload_2hyd
[31]	ADC-A_HC_G0 / 1xPayload,1xPayload_1hyd,1xPayload_2hyd
[32]	ADC-A_LC_HC_G0 / 2xPayload_2hyd
[33]	ADC-A_HC_G0 / 1xDeamidation (N),2xOxidation (MW),1xPayload
[34]	ADC-A_HC_1GK / 1xDeamidation (N),1xPayload_2hyd
[35]	ADC-A_HC_G0 / 1xDeamidation (N),1xDeamidation (Q),1xOxidation (MW),1xPayload_2hyd
[36]	ADC-A_LC_HC_G0
[37]	ADC-A_LC_HC_1GK_loss / 1xDeamidation (N),2xPayload_2hyd
[32']	ADC-A_LC_HC_G0 / 2xPayload_2hyd
[38]	ADC-A_LC_HC_G0 / 1xDeamidation (N),2xPayload_2hyd
[39]	ADC-A_HC_G0 / 3xPayload_2hyd

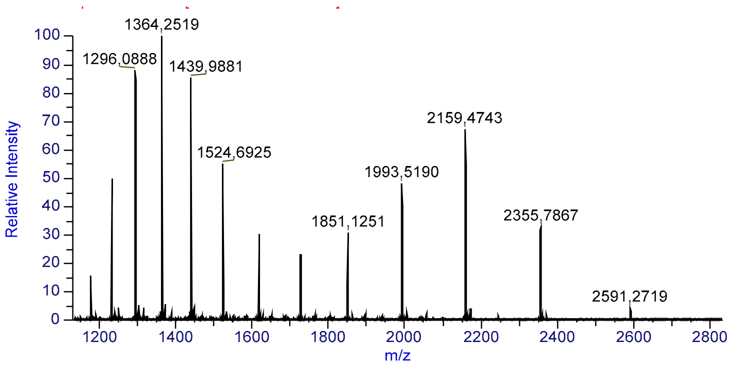

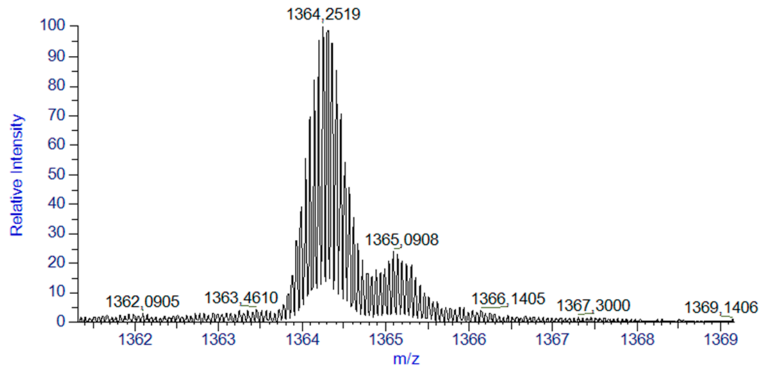

Figure S1. *(Top) Mass spectra of LC DAR 1 at 0h in mouse plasma. (Bottom) Zooming on 1364.25 m/z, which represents a single charge state (+18) of LC conjugated with 1 linker payload (with no hydrolysis). 1365.09 m/z represents a single charge state (+18) of LC conjugated with 1 linker payload (with no hydrolysis and 1 oxidation).*

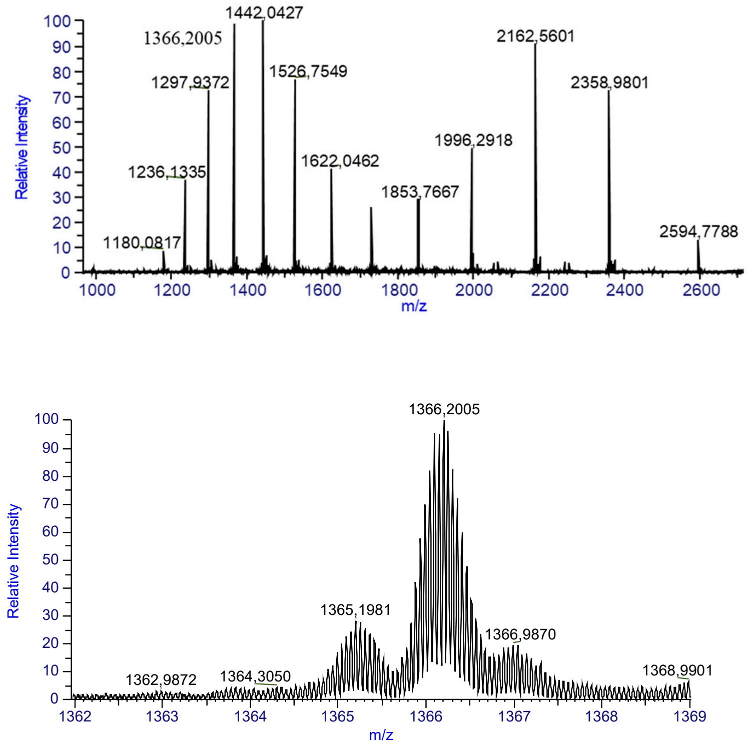

Figure S2. *(Top) Mass spectra of LC DAR 1 with 2 hydrolyses at 168h in mouse plasma. (Bottom) Zooming on 1366.20 m/z, which represents a single charge state (+18) of LC conjugated with 1 linker payload (with 2 hydrolyses). 1365.20 m/z represents a single charge state (+18) of LC conjugated with 1 linker payload (with 1 hydrolysis). 1366.99 m/z represents a single charge state (+18) of LC conjugated with 1 linker payload (with 2 hydrolyses and 1 oxidation).*

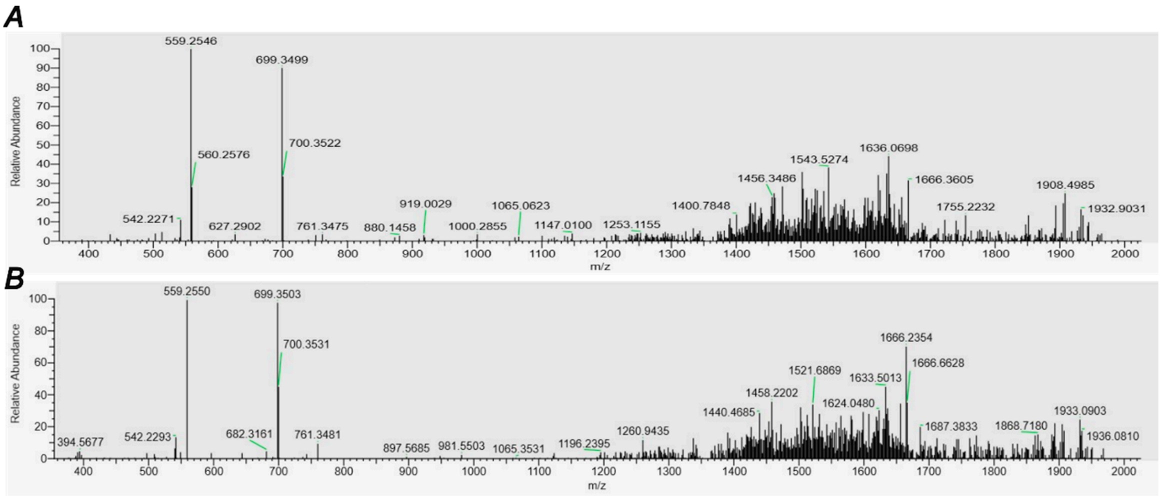

Figure S3. *MS/MS spectra induced with collisional induced dissociation of* LC DAR1 2hyd MS/MS spectrum at 96 and 168h in mouse plasma.

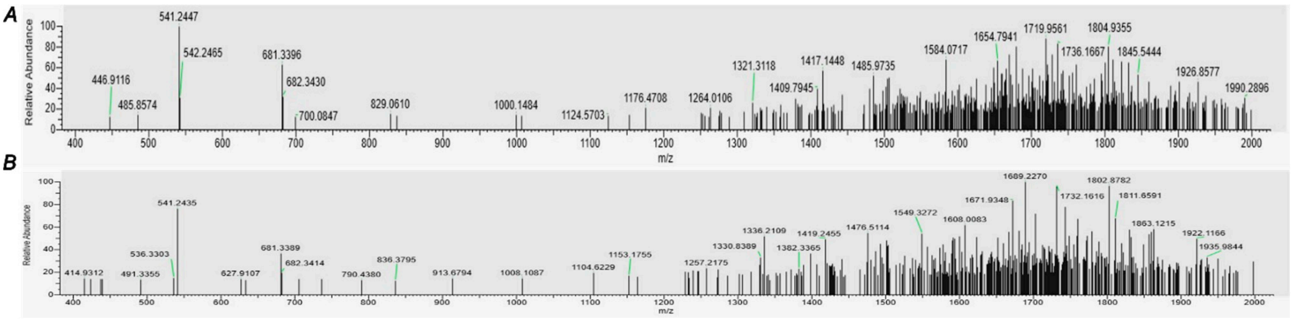

Figure S4. *MS/MS spectra induced with collisional induced dissociation of* ADC-A_HC_G0 / 2xPayload,1xPayload_1hyd MS/MS spectrum at 5 and 24h in mouse plasma.

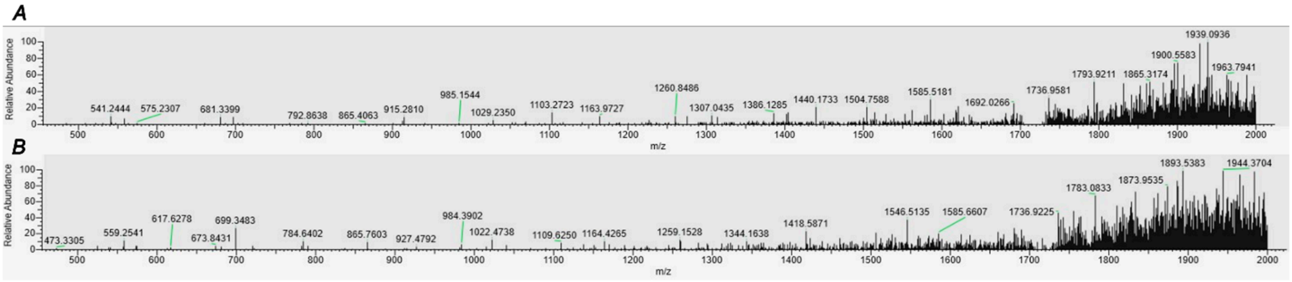

Figure S5. *MS/MS spectra induced with collisional induced dissociation of* LC HC DAR2 G0 2hyd MS/MS spectrum at 48 and 72h in mouse plasma.

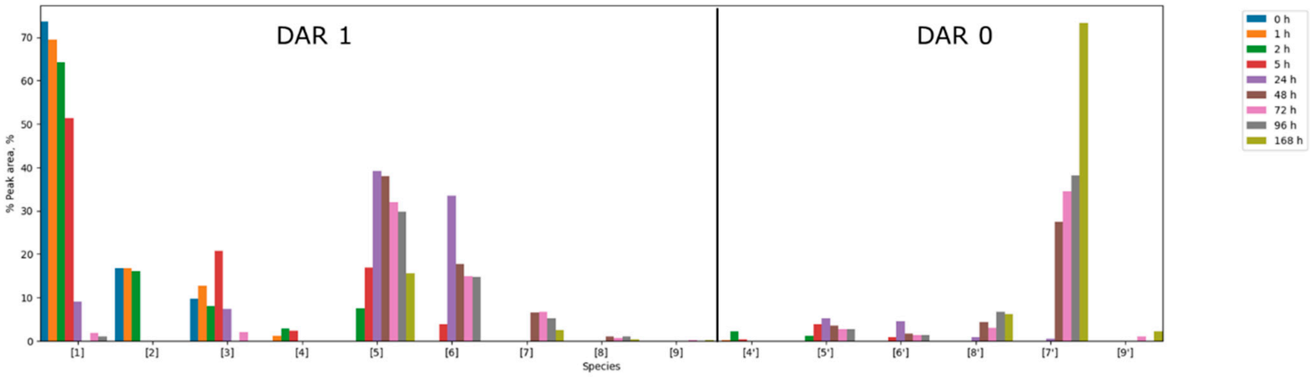

Figure S6. *Intact DAR LC analysis for ADC-A in in vitro stability mouse plasma samples. Relative % of peak area (y-axis) for all identified LC species over time during mouse plasma in vitro stability.*

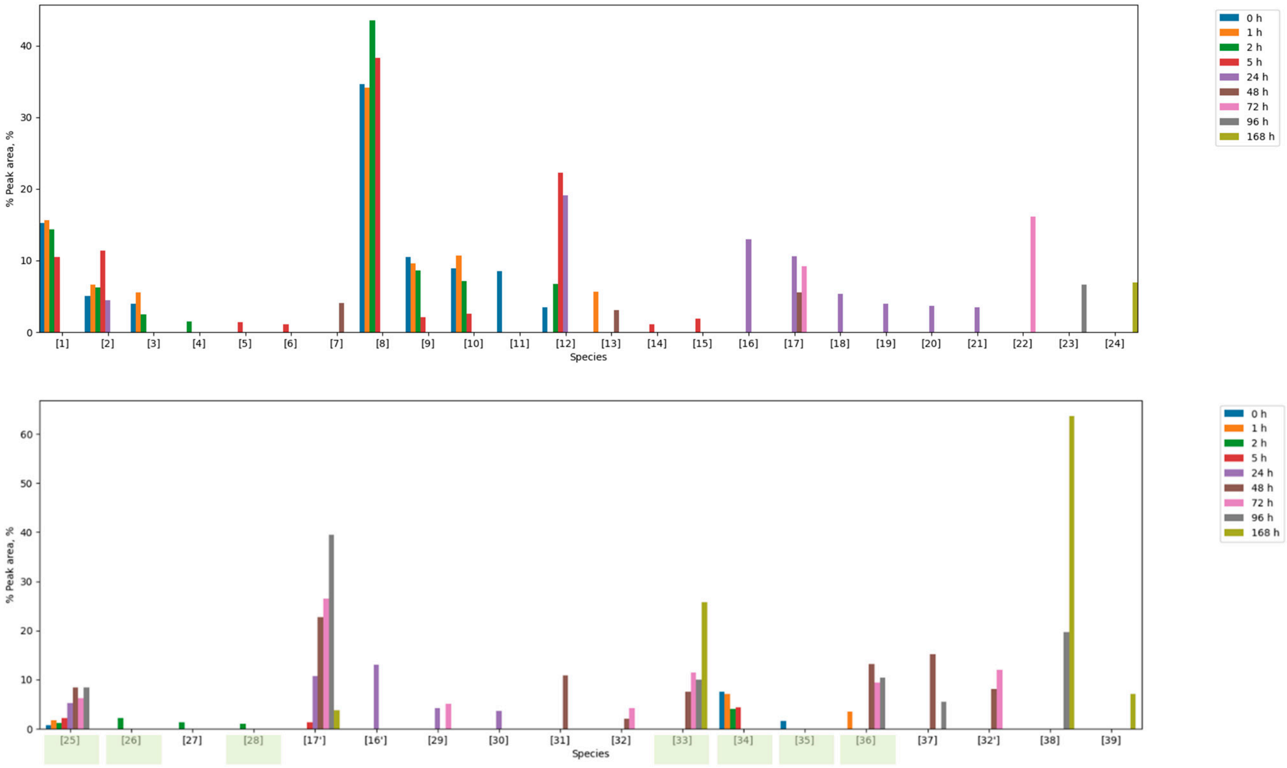

Figure S7. *Intact DAR HC and half-antibody analysis for ADC-A in in vitro stability mouse plasma samples. Retro-Micheal species are highlighted in green. Relative % of peak area (y-axis) for all identified HC and half-antibody species over time during mouse plasma in vitro stability.*
